# Optimizing Digital Cardiac Rehabilitation Using the Multiphase Optimization Strategy: Mixed Methods Feasibility Study

**DOI:** 10.2196/77742

**Published:** 2026-06-09

**Authors:** Eanna Kenny, John William McEvoy, Jenny McSharry, Julie Doyle, Oonagh M Giggins, Lisa Hynes, Andrew W Murphy, Rod S Taylor, Molly Byrne

**Affiliations:** 1 Health Behaviour Change Research Group School of Psychology Ollscoil na Gaillimhe – University of Galway Galway Ireland; 2 School of Medicine Ollscoil na Gaillimhe – University of Galway Galway Ireland; 3 National Institute for Prevention and Cardiovascular Health Galway Ireland; 4 NetwellCASALA Dundalk Institute of Technology Dundalk Ireland; 5 Croí, West of Ireland Cardiac Foundation Galway Ireland; 6 Health Research Board Primary Care Clinical Trials Network Ireland Ollscoil na Gaillimhe – University of Galway Galway Ireland; 7 MRC/CSO Social and Public Health Sciences Unit & Robertson Centre for Biostatistics Institute of Health and Well Being University of Glasgow Glasgow United Kingdom

**Keywords:** acceptability, cardiac rehabilitation, cardiovascular disease, digital, feasibility, multiphase optimization strategy

## Abstract

**Background:**

Cardiac rehabilitation (CR) is an evidence-based, multicomponent intervention. However, participation in and reach of CR remain suboptimal globally. Digital CR is a promising alternative to traditional center-based CR, with the potential to increase intervention reach and efficiency. However, efforts to increase the efficiency of digital CR require an understanding of the relative effectiveness of the components of CR, which is currently lacking. The Multiphase Optimization Strategy provides a framework to evaluate the effects of individual components within complex interventions.

**Objective:**

This mixed methods study explored the feasibility and acceptability of implementing the procedures of a factorial design and delivering multiple intervention components in preparation for an optimization randomized controlled trial of a digital CR intervention.

**Methods:**

Patients attending CR in a community setting were randomized to 1 of 8 experimental conditions in a 2 × 2 × 2 (2^3^) factorial trial design. Each condition received a different combination of three intervention components over a 6-week study period (1) goal setting and self-monitoring, (2) education, and (3) feedback messages. Feasibility was assessed through intervention fidelity (eg, usage statistics) and outcome measure data completeness. Acceptability was measured using the System Usability Scale, a questionnaire, and semistructured interviews based on the Theoretical Framework of Acceptability.

**Results:**

A total of 8 participants were recruited and retained in the study. The mean age was 75 (SD 5.6) years, and the majority were female (5/8, 62.5%). The digital CR intervention demonstrated good usability (System Usability Scale score 72.1, SD 19.1), and 83.3% (5/6) of participants found the digital technology acceptable. However, only half (2/4, 50%) found the feedback messages acceptable. Fidelity was high for goal setting/self-monitoring and feedback but lower for education. Qualitative findings indicated that participants held positive attitudes toward the intervention and reported improvements in physical activity, although many expressed a preference for more tailored feedback and 2-way communication. Of the 3 prespecified progression criteria, usability met the “Go” criterion, whereas intervention fidelity, acceptability, and outcome measure data completeness met the “Amend” threshold.

**Conclusions:**

This study demonstrated the feasibility of implementing a factorial design and delivering multiple intervention components within a digital CR intervention. While the intervention was generally acceptable, modifications to the education and feedback components are necessary prior to conducting a pilot optimization randomized controlled trial.

## Introduction

Cardiovascular disease (CVD) is the leading cause of death worldwide [1]. Environmental and lifestyle risk factors significantly contribute to the development of CVD, and individuals diagnosed with CVD benefit from interventions that aim to reduce cardiac risk factors and improve secondary prevention. Cardiac rehabilitation (CR) is a secondary prevention program designed to stabilize, slow, or reverse the progression of CVD while enhancing participants’ functional status and quality of life [2]. Despite international clinical guidelines recommending that CR should be provided for all individuals with CVD [3], global participation rates remain low, with fewer than 50% of those who are eligible enrolling in most countries [4]. Additionally, CR capacity continues to be significantly constrained [4,5], and increasing survival rates have led to higher demand for CR [6]. Consequently, alternative models of CR are needed to increase participation and capacity [7]. Digitally delivered CR is one emerging model that has the potential to enhance intervention reach and improve the efficiency of service delivery [2,7].

CR is a complex, multicomponent intervention, and improving its accessibility and effectiveness requires a granular understanding of which components contribute meaningfully to improving outcomes. Identifying effective components allows for the removal of inactive elements, resulting in a more efficient and condensed intervention. However, our understanding of the effective components of CR remains unclear, and defining the optimal content of CR interventions has been identified as a major challenge [8]. Existing trials of CR have primarily used single-arm or parallel group randomized designs, which are suitable for evaluating complex interventions as a whole against controls but provide limited information about the relative contributions of each intervention component. Alternative trial designs are needed to determine the individual effects of each component and therefore improve the overall effectiveness of these interventions.

The Multiphase Optimization Strategy (MOST) is a methodological framework for optimizing behavioral interventions to improve intervention effectiveness, efficiency, and scalability [9]. MOST involves 3 phases: preparation, optimization, and evaluation. In the preparation phase, the objective is to develop a conceptual model of the intervention and identify candidate intervention components. In the optimization phase, the performance of the candidate intervention components is evaluated in an optimization randomized controlled trial (RCT), using an efficient experimental design. Factorial designs are commonly used in this phase as they allow each candidate intervention component to be set at different levels (eg, present vs absent), allowing all possible combinations of the components to be tested. The results provide estimates for the main and interaction effects of each component, thereby informing the composition of the most effective intervention package. Finally, in the evaluation phase, the optimized intervention package is evaluated in what is referred to within MOST as an evaluation RCT.

As part of the preparation phase of MOST, we developed a conceptual model of digital CR. The model is based on the Capability, Opportunity, Motivation, and Behavior (COM-B) model [10] and the Theoretical Domains Framework (TDF) [11,12], and was developed through a synthesis of evidence from a systematic review of digital CR interventions [13], a qualitative study with patients who completed digital CR [14], and a review of international guidelines [15]. The conceptual model specifies the candidate intervention components, behavior change techniques (BCTs), targeted theoretical domains and COM-B factors, and the behavioral and clinical outcomes of interest. A recommended next step in the preparation phase is to pilot-test the candidate intervention components and the optimization trial procedures. Pilot testing helps assess the feasibility and acceptability of each intervention component and various aspects of the study design, such as recruitment, randomization, and the selection of outcome measures [9]. This step provides valuable information for refining the components and study protocol before proceeding to the optimization phase.

**Figure 1:**
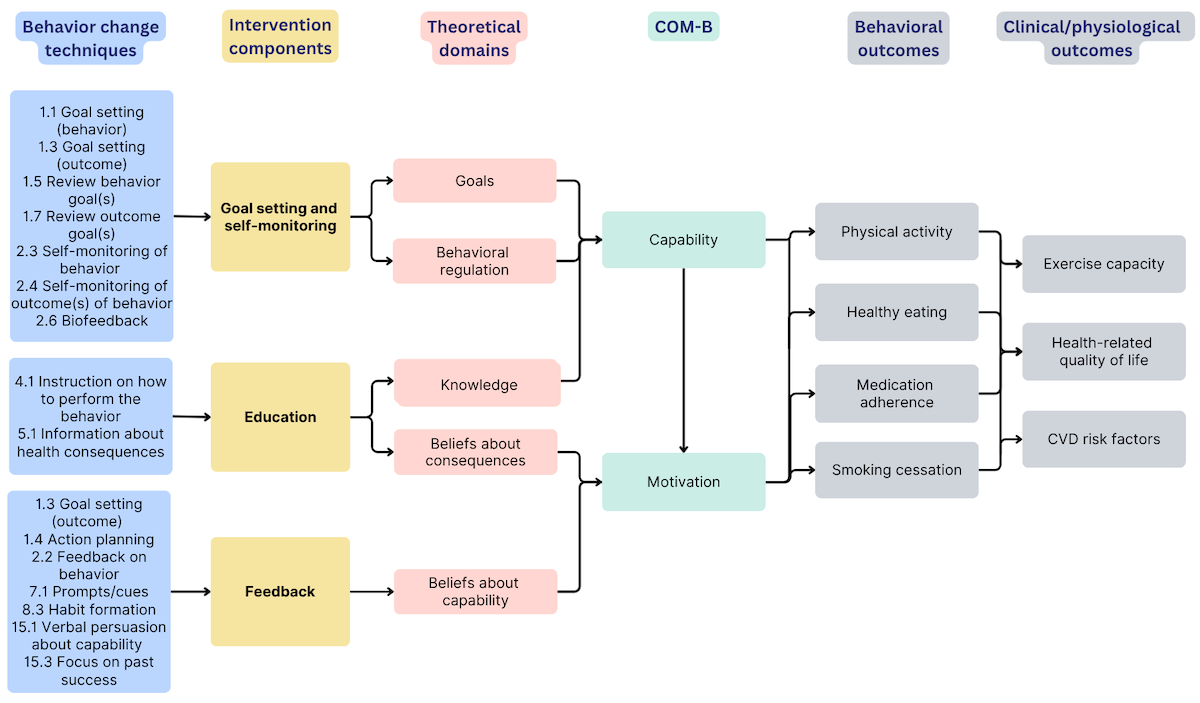
Conceptual model of the digital cardiac rehabilitation intervention. COM-B: Capability, Opportunity, Motivation, and Behavior; CVD: cardiovascular disease.

Due to time and resource constraints, it was not feasible to develop a novel digital CR intervention to meet the specifications of the conceptual model. Instead, we pragmatically identified existing digital health technologies capable of delivering the intervention components. Two such technologies, ProACT (NetwellCASALA) and Textmagic, were selected, as they could deliver 3 of the 5 intervention components specified in the model: goal setting and self-monitoring, education, and feedback. ProACT is a digital health platform designed to support older adults’ self-management of chronic conditions [16]. It integrates health data from various sensing devices (eg, Withings ScanWatch and Withings BPM Connect) into a web-based application (ProACT CareApp), through which participants can set goals, track health data, and access educational content. The platform was developed using a user-centered design approach, incorporating co-design sessions and usability testing to inform its development and refinement [16]. ProACT was used to deliver the goal setting and self-monitoring and education components. Textmagic, a text messaging service that allows scheduling SMS text message delivery and 2-way communication, was used to deliver the feedback component. These 3 components were selected for inclusion in this study because they were the least resource-intensive and could be delivered entirely digitally, without requiring assistance from CR staff (eg, physiotherapists or cardiovascular nurses). Figure 1 presents a conceptual model illustrating the 3 intervention components and their hypothesized impact on outcomes. While this study does not include all components identified in our preliminary work, it provides valuable insights into the feasibility and acceptability of select intervention components and aspects of the study design.

Therefore, the aim of this study was to continue the preparatory phase of MOST by exploring the feasibility and acceptability of implementing the procedures of a factorial design and delivering multiple intervention components in preparation for an optimization RCT of a digital CR intervention. The specific objectives of the study were to assess (1) the usability of the digital technology; (2) the fidelity of the intervention components; and (3) the acceptability of the study design, procedures, outcome measures, and intervention components.

## Methods

### Ethical Considerations

This study is reported in accordance with the extended CONSORT (Consolidated Standards of Reporting Trials) checklist of information to include when reporting a pilot or feasibility trial (Multimedia Appendix 1) [17], the Template for Intervention Description and Replication (TIDieR) checklist (Multimedia Appendix 2) [18], and the MOST Preparation Reporting (MOST PREP-REP) checklist (Multimedia Appendix 3) [19]. Ethical approval for this study was granted by the relevant University Research Ethics Committee (reference number: 2023.06.008). A data protection impact assessment was completed for this study and reviewed by the data protection officer at the relevant university. Additionally, a data processing agreement and a research evaluation agreement were put in place between the data controller and the proprietors of ProACT. Interested participants received detailed information about the study, and informed consent was obtained electronically via Qualtrics [20] or through a hard copy before the study commenced. Recruitment took place in April and May 2024.

### Study Design

This was an exploratory mixed methods feasibility study of a 2 × 2 × 2 (2^3^) factorial trial testing 3 intervention components of a digital CR intervention. Participants were randomly assigned to 1 of 8 experimental conditions (Table 1). Each condition comprised a unique combination of the 3 intervention components, each of which had 2 levels. The components and levels included were: (1) goal setting and self-monitoring (yes vs no), (2) education (yes vs no), and (3) feedback (yes vs no). These components are described in detail below.

**Table 1:** Experimental conditions in the 2 × 2 × 2 (23) factorial design.

Experimental condition	Goal setting and self-monitoring	Education	Feedback	Participant allocation
1	Yes	Yes	Yes	P001
2	Yes	Yes	No	P002
3	Yes	No	Yes	P005
4	Yes	No	No	P006
5	No	Yes	Yes	P007
6	No	Yes	No	P003
7	No	No	Yes	P008
8	No	No	No	P004

### Participants and Recruitment

Participants were attending Phase IV CR at Croí, a community-based heart and stroke charity in Galway, Ireland. The Croí Phase IV CR program is a rolling, 6-week exercise program led by a cardiac physiotherapist/physical activity specialist for individuals who have completed a structured, supervised Phase III CR program. Participants were eligible for inclusion if they: (1) were adults aged 18 years or older, (2) were attending Croí for Phase IV CR, and (3) owned a smartphone. Participants were excluded if they had a cardiac pacemaker or other internal medical device. This is because the Withings ScanWatch, which was used as part of the study intervention, radiates electromagnetic fields that may interfere with other medical devices, such as pacemakers. Participant recruitment was facilitated by study partners at Croí, who contacted eligible participants via email with study details and an invitation to participate. Additional recruitment strategies included visual advertisements and an informational booth at Croí, where a research team member was available to provide further information.

### Intervention

The digital CR intervention was delivered using ProACT and Textmagic, as outlined in the Introduction section. All participants continued to attend their usual Phase IV CR exercise classes during the study period, which served as a constant background condition across all experimental conditions. Figure 1 illustrates the conceptual model of the intervention, and the included components are described in detail below.

### Intervention Components

#### Goal Setting and Self-Monitoring

Participants were instructed to set behavioral goals (eg, physical activity targets) and outcome goals (eg, target weight) via the ProACT CareApp. To facilitate self-monitoring, participants received 2 off-the-shelf smart wearable devices: the Withings ScanWatch for tracking heart rate and physical activity, and the Withings BPM Connect, a clinically validated blood pressure monitor for home use [21]. The devices transmitted data via Bluetooth to the Withings Health Mate app, which was synchronized with the ProACT CareApp. Participants could track their progress and change their goals at any time. This component encompassed the following BCTs: 1.1 goal setting (behavior), 1.3 goal setting (outcome), 1.5 review behavior goal(s), 1.7 review outcome goal(s), 2.3 self-monitoring of behavior, 2.4 self-monitoring of outcome(s) of behavior, and 2.6 biofeedback. This component was designed to assist patients in creating clear mental representations of their desired outcomes while also providing a way of objectively managing their actions toward the attainment of their goals.

#### Education

The education component included information on CVD, managing risk factors, and leading a healthy lifestyle. The materials were presented in the form of articles and videos and were delivered via the ProACT CareApp. This component included the BCTs 4.1 instruction on how to perform the behavior and 5.1 information about health consequences and aimed to enhance participants’ knowledge and beliefs about the consequences of their behavior in the context of CVD. It was hypothesized that targeting these theoretical domains would increase participants’ perceived capability and motivation to improve health-related behaviors (eg, physical activity, healthy eating, medication adherence, and smoking cessation).

#### Feedback

Participants received general feedback via SMS text messages designed to increase their beliefs about their capability to manage their condition and perform the required health-related behaviors (eg, physical activity, healthy eating, and medication adherence). The content of the SMS text messages was based on previous studies that aimed to promote behavior change among patients attending CR [22,23]. Each message contained 1 of 7 BCTs: 1.3 goal setting (outcome), 1.4 action planning, 2.2 feedback on behavior, 7.1 prompts/cues, 8.3 habit formation, 15.1 verbal persuasion about capability, and 15.3 focus on past success. Participants received 3 messages per week over the course of the 6 weeks (18 text messages in total). SMS text messages were personalized to include the participant’s name but otherwise were standardized and sent in a set order using Textmagic. Participants were able to opt out of receiving the messages at any time. Examples of the messages are provided in Multimedia Appendix 4.

### Procedure

Participants completed a baseline and follow-up questionnaire either electronically via Qualtrics [20] or on paper. The baseline questionnaire captured demographic data (age, gender, ethnicity, marital status, employment, and education), clinical history (heart condition, medication use, and Phase III CR attendance), and information on health behaviors, clinical outcomes, and psychological mediators (see below). Following completion of the baseline questionnaire, participants were randomly assigned to 1 of 8 experimental conditions, determining which intervention components they received. Participants had an equal chance of allocation to each group. Participants allocated to the goal setting and self-monitoring or education components were invited to an introductory face-to-face session at Croí where they received the ProACT CareApp on their smartphone, devices (eg, Withings ScanWatch and Withings BPM Connect), a device instruction manual, and training on using the technology. Participants were instructed to use the app and devices as frequently as they wished. At the end of the 6-week study period, all participants were invited to complete a follow-up questionnaire and a semistructured, one-to-one interview about their experience. The interviews were audio recorded and transcribed verbatim. The average interview duration was 15 (range 6-25) minutes.

### Outcomes

The primary outcomes of this study related to feasibility and acceptability. Feasibility was assessed by tracking the completeness of outcome measure data and intervention fidelity. Fidelity was evaluated across 3 dimensions: delivery, receipt, and enactment [24]. Fidelity of delivery was evaluated by recording the SMS text messages sent to participants. Fidelity of receipt was measured by tracking data uploads and the number of educational materials viewed on the ProAct platform. Fidelity of enactment was explored through the semistructured interviews, which examined how participants incorporated the intervention components into their everyday lives.

Acceptability was measured using the System Usability Scale (SUS) [25], a 10-item questionnaire scored on a 5-point Likert scale ranging from 1 (strongly agree) to 5 (strongly disagree). The SUS produces a composite score ranging from 0-100, with a score greater than 68 considered above average [26]. Additional data on acceptability were collected through a questionnaire and semistructured interviews informed by the Theoretical Framework of Acceptability (TFA) [27]. The TFA conceptualizes acceptability across 7 constructs: affective attitude, burden, perceived effectiveness, ethicality, intervention coherence, opportunity costs, and self-efficacy (Table 2). A TFA-based questionnaire was developed and adapted for this study. The acceptability of the digital technology (eg, smartphone apps, smartwatches, and blood pressure monitor) was evaluated across 6 constructs: affective attitude, burden, perceived effectiveness, opportunity costs, intervention coherence, and self-efficacy. The acceptability of the SMS text messages was assessed across 3 constructs: affective attitude, perceived effectiveness, and intervention coherence. The semistructured interviews further explored participants’ experiences with the study procedures, intervention components, and data collection tools.

**Table 2:** The Theoretical Framework of Acceptability construct definitions [27].

Construct	Definition
Affective attitude	How an individual feels about the intervention
Burden	The perceived amount of effort that is required to participate in the intervention
Ethicality	The extent to which the intervention has good fit with an individual’s value system
Intervention coherence	The extent to which the participant understands the intervention and how it works
Opportunity cost	The extent to which benefits, profits, or values must be given up to engage in the intervention
Perceived effectiveness	The extent to which the intervention is perceived as likely to achieve its purpose
Self-efficacy	The participant’s confidence that they can perform the behavior(s) required to participate in the intervention

The feasibility of the data collection tools intended for use in a future optimization trial was also assessed. Traditionally, optimization RCTs use a single primary outcome; however, recent advances in decision-making approaches in MOST support the inclusion of multiple outcomes [28,29]. Therefore, we explored the feasibility and acceptability of capturing behavioral and clinical outcomes, as well as hypothesized mediators of change at baseline and 6 weeks. Physical activity was assessed using the International Physical Activity Questionnaire [30]. Healthy eating behaviors were evaluated with the Mediterranean Diet Scale [31], while medication adherence was measured using the Adherence to Refills and Medications Scale-7 [32]. Smoking cessation was recorded via self-report, and health-related quality of life was measured using the EQ-5D [33]. Anxiety and depression levels were assessed using the Hospital Anxiety and Depression Scale [34], and self-efficacy was measured using the Cardiac Self-Efficacy Scale [35]. CVD knowledge and risk perception were assessed using the Attitudes and Beliefs about Cardiovascular Disease Knowledge and Risk Questionnaire [36]. Finally, goals and behavioral regulation were evaluated with an adapted version of the TDF questionnaire [37].

### Sample Size

A total of eight participants were recruited for the study, allowing for one participant per experimental condition. Although a larger sample was initially planned, significant delays and slow recruitment at the single study site made it unfeasible to recruit and collect data on a larger sample within the study’s timeframe. It should be noted that with one participant per condition, no meaningful factorial analysis of component effects or interactions was possible. The purpose of the factorial structure at this stage was to test randomization, allocation, and delivery procedures in preparation for a future, adequately powered optimization trial.

### Progression Criteria

In line with relevant guidance [38], the study team developed prespecified ‘Go/Amend/Stop’ criteria to determine if progression to an optimization RCT was warranted. These criteria were based on recommendations in the literature on cut-off scores for validated scales of usability [26], and incompleteness of outcome data [39,40]. Criteria on acceptability was assessed using qualitative judgement only. Quantitative thresholds were not pre-specified for this criterion as there are no established benchmarks in the digital CR literature for what constitutes an acceptable level of component acceptability. The 3 progression criteria are given in Textbox 1.

**Prespecified progression criteria.**

**Usability of the digital technology**

- Will the digital technology receive a mean SUS score of >68?
- Interpretation of qualitative data relating to usability.

**Acceptability of intervention components**

- Interpretation of qualitative data relating to the acceptability of intervention components.

**Feasibility and acceptability of outcome measures**

- Is data completeness ≥95%?
- Interpretation of qualitative data relating to acceptability of outcome measures.

### Randomization and Blinding

After completing baseline measures, participants were randomly assigned to 1 of the 8 experimental conditions by the lead researcher (EK). Randomization was concealed and performed using a computer-generated randomization scheme. Blinding was not possible as the lead researcher delivered the face-to-face training session to the participants. Participants were not blinded to the experimental condition to which they were allocated.

### Data Analysis

#### Quantitative Data Analysis

Quantitative data were analyzed using SPSS software (version 29; IBM Corp). Descriptive statistics were used to describe the participant sample and to report all primary feasibility and acceptability outcomes. Given the small sample size (n=8), no inferential statistics were performed because the study was not powered to detect differences between groups.

#### Qualitative Data Analysis

Qualitative data were analyzed using the Framework Method [41]. This involved familiarization with the data, coding interview transcripts, applying an analytical framework to index the data, charting the data into a framework matrix, and interpreting the data [41]. The TFA [27] was selected as the analytical framework, and interview data were deductively coded to its 7 constructs. Qualitative data management and analysis were conducted using NVivo (version 14; Lumivero).

### Public and Patient Involvement

A Public and Patient Involvement panel at the Croí heart and stroke charity, consisting of 6 individuals who are living with CVD, was embedded in this research from the outset. The Public and Patient Involvement panel was involved in the preparatory work that informed the development of the conceptual model.

## Results

### Overview

A total of 8 participants were recruited and retained in the study. The mean age was 75 (SD 5.6) years, and the majority were female (5/8, 62.5%). All participants were White (Irish) and retired. Regarding CVD history, 12.5% (1/8) had undergone coronary artery bypass surgery, 37.5% (3/8) had undergone percutaneous transluminal coronary angioplasty, and 25% (2/8) were unsure of their diagnosis. Most participants (7/8, 87.5%) had experienced their cardiac event more than 2 years earlier, and 87.5% (7/8) had attended Phase III CR. Among them, 50% (4/8) attended all sessions, 25% (2/8) attended more than half, and 12.5% (1/8) attended approximately half. None were current smokers. Table 3 presents the characteristics of the sample.

**Table 3:** Demographic and clinical characteristics of the participants (n=8).

Characteristics		Value
**Sex, n (%)**		
	Female	5 (62.5)
Age (years), mean (SD); range		75 (5.5); 67-81
**Ethnicity, n (%)**		
	White	8 (100)
**Education, n (%)**		
	Completed secondary school	1 (12.5)
	Vocational or similar	1 (12.5)
	Some university but no degree	2 (25)
	Third level education	3 (37.5)
	Prefer not to say	1 (12.5)
**Employment status, n (%)**		
	Retired	8 (100)
**Marital status, n (%)**		
	Married	4 (50)
	Living with partner	1 (12.5)
	Widowed	2 (25)
	Never been married	1 (12.5)
**CVD^a^ condition, n (%)**		
	Coronary artery bypass surgery	1 (12.5)
	Percutaneous transluminal coronary angioplasty	3 (37.5)
	Do not know	2 (25)
**Time since cardiac event, n (%)**		
	In the past 6 months	1 (12.5)
	More than 2 years ago	7 (87.5)
Attended Phase III CR^b^, n (%)		7 (87.5)
**Number of sessions attended, n (%)**		
	About half	1 (12.5)
	More than half	2 (25)
	All	4 (50)
Current smoker		0 (0)

^a^CVD: cardiovascular disease.

^b^CR: cardiac rehabilitation.

### Primary Outcomes

#### Usability and Acceptability

The overall mean SUS score for the intervention was 72.1 (SD 19.1). Based on the Sauro-Lewis curved grading scale [42], a score of 68 is considered average (grade C), indicating that the usability of the technology in this study was rated as C+.

Overall, the digital technology (eg, smartphone apps, smartwatch, and blood pressure monitor) used for goal setting and self-monitoring and education was rated as highly acceptable, with 83.3% (5/6) of participants finding it completely acceptable and 16.6% (1/6) considering it completely unacceptable. For the SMS text messages, half of the participants (2/4, 50%) found them completely acceptable, while the remaining 50% (2/4) had no opinion. Table 4 presents the acceptability scores for digital technology and SMS text messages across each TFA construct.

**Table 4:** Acceptability of the digital cardiac rehabilitation intervention.

Survey domain		Survey item	Positive response^a^, n (%)	Negative Response^b^, n (%)	No opinion, n (%)
**Acceptability of the technology (n=6)**					
	Affective attitude	Did you like or dislike the technology used in this study?	5 (83.3)	0 (0)	1 (16.6)
	Burden	How much effort did it take to engage with the technology?	6 (100)	0 (0)	0 (0)
	Intervention coherence	It is clear to me how the technology will help improve my health.	4 (66.6)	0 (0)	2 (33.3)
	Opportunity costs	Using the technology would interfere with my other priorities.	0 (0)	6 (100)	0 (0)
	Perceived effectiveness	The technology has improved my healthy behaviors.	4 (66.6)	0 (0)	2 (33.3)
	Self-efficacy	How confident did you feel about using the technology?	5 (83.3)	0 (0)	1 (16.6)
	General acceptability	How acceptable was the technology to you?	5 (83.3)	1 (16.6)	0 (0)
**Acceptability of the SMS text messages (n=4)**					
	Affective attitude	Did you like or dislike the text messages in this study?	1 (25)	1 (25)	2 (50)
	Intervention coherence	It is clear to me how the text messages will help improve my health.	1 (25)	1 (25)	2 (50)
	Perceived effectiveness	The text messages have improved my healthy behaviors.	2 (50)	1 (25)	1 (25)
	General acceptability	How acceptable was receiving the text messages for you?	2 (50)	0 (0)	2 (50)

^a^Positive response categories: (strongly) like, no effort or a little, (strongly) agree, (very) confident, or (completely) acceptable, as applicable.

^b^Negative response categories: (strongly) dislike, huge effort or a lot, (strongly) disagree, (very) unconfident, or (completely) unacceptable, as applicable.

#### Acceptability of the Digital Technology

Among the 6 participants who received the digital technology, most (5/6, 83.3%) liked or strongly liked it, while 16.6% (1/6) disliked it. The technology was not perceived as burdensome, with all participants reporting that it required little to no effort to use. Confidence in using the technology was high, with 83.3% (5/6) feeling confident or very confident, while 16.6% (1/6) had no opinion. Perceived effectiveness of the technology varied, with 66.6% (4/6) agreeing or strongly agreeing that it improved their healthy behaviors, while 33.3% (2/6) had no opinion. Similarly, 66.6% (4/6) of participants agreed or strongly agreed that the role of technology in supporting health improvement was clear, while 33.3% (2/6) had no opinion. There was no perceived opportunity cost associated with using the technology, as all participants reported that it did not interfere with other priorities.

#### Acceptability of the SMS Text Messages

Acceptability of the SMS text messages was mixed. Among the 4 participants who received them, 25% (1/4) strongly liked the messages, while an equal proportion disliked them. The remaining half (2/4, 50%) had no opinion. Perceived effectiveness was moderate, with 50% (2/4) agreeing or strongly agreeing that the messages improved their healthy behaviors, while 25% (1/4) disagreed and 25% (1/4) had no opinion. Similarly, 25% (1/4) agreed or strongly agreed that the purpose of the messages in supporting health improvement was clear, whereas 25% (1/4) disagreed and 50% (2/4) had no opinion.

### Feasibility of Study Procedures

All participants were successfully randomized to 1 of 8 experimental conditions using a computer-generated randomization sequence. Face-to-face onboarding sessions at Croí were completed for all participants assigned to intervention components involving digital technology. Each participant received the appropriate materials, including devices and manuals, and installation of the ProACT CareApp and Withings Health Mate app was completed without major technical issues. Monitoring of technology usage confirmed that all digital components were delivered to the correct groups in accordance with the study design. All study procedures were implemented as planned, with no protocol deviations observed.

### Feasibility of Outcome Measures

All 8 participants who completed baseline measures also completed the follow-up assessment. Data completeness for outcome measures was high, with 92.6% complete at baseline (T1) and 90.6% at follow-up (T2). The Cardiac Self-Efficacy Scale had the highest proportion of missing data at both time points, (15.4% at T1 and 34.6% at T2). Other measures with more than 10% missing data included the International Physical Activity Questionnaire (11.4% at T1 and T2), the Adherence to Refills and Medications Scale-7 (12.5% at T1), the Attitudes and Beliefs about Cardiovascular Disease Risk Questionnaire (20.3% at T2), and the TDF questionnaire (14% at T2).

### Intervention Fidelity

Participants who received the ProACT CareApp used it for a mean of 22.2 (SD 20.6) days. Engagement was primarily driven by the goal setting and self-monitoring component. Among those who received this component, step data were uploaded for a mean of 41.5 (SD 6.5) days, blood pressure readings were recorded on a mean of 14.3 (SD 13.4) days, and weight was recorded on 3.8 (SD 3.1) days. One participant (P002) did not regularly access the ProACT CareApp, which resulted in blood pressure data not being transferred. Fidelity to the education component was low, with participants viewing educational materials on a median of 2.5 (IQR 1.5-3.5, range 1-4) days. The number of distinct educational items viewed ranged from 2 to 20 (median 3.5, IQR 2.5-12). In contrast, fidelity to the feedback component was high; all SMS text messages were successfully delivered, and no participants opted out of receiving them. Table 5 provides a detailed summary of the ProACT CareApp usage statistics for each participant.

**Table 5:** Usage statistics of the ProACT CareApp for each participant.

Metric	P001	P002	P003	P005	P006	P007
ProACT CareApp used (days), n	46	3	5	46	28	5
Steps uploaded (days), n	49	39	—^a^	44	34	—
Blood pressure uploaded (days), n	30	0	—	20	7	—
Weight recorded (days), n	8	1	—	2	4	—
Education viewed (days), n	3	1	4	—	—	2
Distinct education items viewed, n	3	2	20	—	—	4

^a^Indicates that the participant did not use the corresponding feature.

### Qualitative Findings

All 8 participants took part in a semistructured interview to discuss their experience following the completion of the study.

#### Affective Attitude

Overall, participants expressed positive attitudes toward the intervention. Many found it beneficial, with 1 participant stating, “‘I’m delighted I did it and I feel it did me good” (P006, female, aged 80 years). One participant initially felt apprehensive about using the technology, fearing that he might damage the equipment. However, he later described how the technology quickly became part of his routine and ultimately provided reassurance:

It just became routine after a while and surprisingly, comforting. Which I didn’t expect, that I was kind of keeping a check – ‘Oh yeah, that’s not too bad, my blood pressure’s not too bad’. Yeah, there was a comforting element that I hadn’t expected.

P001, male, aged 70 years

Despite these positive experiences, not all participants had favorable perceptions. Some reported challenges with the BPM Connect device used for blood pressure monitoring. One participant described how regular use of the device led to physical discomfort and stress:

And the stress was coming from well A, having to find time to do it; B, the pain of it, the soreness of it and not being 100% sure had I it on correctly. Then, my blood pressure being high.

P005, female, aged 67 years

Attitudes toward the SMS text messages were similarly mixed. While some participants reported “no problem with them” (P008, male, aged 74 years), others found them irritating or irrelevant. One participant expressed frustration, stating:

Whereas the messages were just random and you know, patronising and irrelevant and you’d want to throw a few expletives at it.

P005, female, aged 67 years

#### Burden

Most participants did not find the study procedures, outcome measures, or intervention components burdensome. As 1 participant stated, “It was no imposition at all from beginning to end, quite honestly” (P001, male, aged 70 years). However, some found certain aspects inconvenient. One participant highlighted the effort required to use the BPM Connect device compared to a smartwatch:

With the watch, you just put it on and off you went, whereas with that [BPM Connect], you had to set time aside. You had to, you know, take off clothes so that you had bare skin for it. And with the cold weather, that became a nuisance, and having to set time aside became a nuisance.

P005, female, aged 67 years

An unintended consequence of participating was also reported by 1 participant, who experienced muscle cramps due to a sudden increase in physical activity: “the main thing is not to overdo it, I suppose. I did walk too fast and one day, I had cramps, that night” (P006, female, aged 80 years).

#### Intervention Coherence

Most participants reported that they understood the intervention and recognized how it could lead to improvements in healthy behaviors, particularly in relation to goal setting and self-monitoring. One participant, using such a smartwatch for the first time, remarked, “I can see how it would be enormously beneficial to overall health, not just heart health’ (P005, female, aged 67 years). Similarly, another participant emphasized the value of blood pressure monitoring in identifying potential complications: “it’s a good way of detecting if there’s complications due to arise” (P002, female, aged 70 years). A third participant highlighted the advantage of being able to share blood pressure readings with their general practitioner at check-ups, viewing this form of monitoring as beneficial for both themselves and their general practitioner. They further stated that such monitoring technology has the potential to save lives: “I have no doubt there will be lives saved as a result of this technology” (P001, male, aged 70 years).

#### Perceived Effectiveness

Most participants reported positive experiences with the intervention and believed it encouraged healthy behaviors. The goal setting and self-monitoring component was particularly motivating:

I found it very positive, you know. It was good for me as well and I felt, I mean I wouldn’t ever miss a day without doing it which is good whereas if I wasn’t doing the thing [the study], I might say, ‘Ah sure I’ll leave it a day’. I’m not terribly motivated.

P006, female, aged 80 years

Participants also described how setting physical activity goals encouraged them to be more active:

So if you’re walking along and you’re thinking, okay I’m going to go in here now because I’m finished walking, you’d look at this and if it was coming up to 100%, you’d go, arra I’ll walk another bit and try and get it up past. And so, that happened a lot. So I was walking extra steps to get to the goal or to get to the double goal. And sometimes the triple.

P005, female, aged 67 years

Regarding the educational materials, participants reported that they were informative and served as a reminder about cardiovascular health: “It made me, you know, think more deeply about some aspects of cardiovascular in particular” (P003, male, aged 81 years). Perceptions of the effectiveness of the SMS text messages varied. Some participants found them useful as reminders, particularly for medication adherence:

I was up in [location] and I forgot to take my medicines because I was out of my normal routine. It reminded me and I took my medicines, which is good.

P008, male, aged 74 years

For others, the messages acted as a prompt for physical activity, “a little bit like your conscience reminding you” (P007, female, aged 80 years).

However, many participants found the messages unnecessary or irrelevant. One participant commented, “they weren’t necessary to buck me up, if you like. I was already motivated” (P001, male, aged 70 years). Participants also questioned the relevance of the messages, describing them as too general:

They just seemed to be random messages coming in out of nowhere that, you know, a lot of them didn’t have any relevance to me so I didn’t look at them.

P005, female, aged 67 years

One participant suggested that greater personalization or a 2-way communication system would enhance this intervention component:

It’s only a one way system...when something cropped up again, I thought, ‘Well he’s no idea. The last day he said this, I did 15,000 steps and then he’s saying increase your exercise’. Now, that doesn’t matter but do you know, I just thought it was too one sided for my complete participation.

P007, female, aged 80 years

#### Self-Efficacy

Most participants reported few difficulties or challenges using the technology. The goal setting and self-monitoring component was described as intuitive, “easy to follow and easy to use” (P002, female, aged 70 years). Similarly, the educational materials were also generally considered accessible, “the app was ideal, it wasn’t a bit complicated. It was simple. Easy to understand” (P003, male, aged 81 years). However, 1 participant reported struggling with the educational materials:

I looked at it once and I said, ‘In the name of God, what’s this all about’, and I still don’t know what it’s all about. What was it all about? I mean, it had a list of, I can’t remember rightly, I only looked at it the once. It had headings and I didn’t know what I was supposed to do with them. You see because I don’t use apps.

P007, female, aged 80 years

When it was explained that the headings contained articles and videos on CVD, the participant noted that they would have preferred to view these materials on a computer rather than a phone, as they found it easier to read on a larger screen.

Furthermore, some participants experienced difficulties when recording blood pressure readings from the BPM Connect in the ProACT CareApp. While the readings were intended to transmit wirelessly from the device to the app, this process was not always reliable, as evidenced by 1 participant not transferring any data. When automatic transmission failed, participants had to enter their readings manually. However, 1 participant observed that it was not possible to manually enter a blood pressure reading for a previous day or to edit an incorrectly entered reading, highlighting a lack of flexibility in the system.

Finally, 3 participants encountered technical difficulties when completing the baseline assessment on Qualtrics. One participant described the issue: “I was stuck at a point there. I couldn’t, it just wouldn’t let me past that” (P008, male, aged 74 years). A paper-based questionnaire was offered to these participants as an alternative.

### Progression Criteria

Of the predefined progression criteria, two met the ‘Go’ criterion (retention of participants, and usability of the digital technology), while two met the ‘Amend’ criterion (acceptability of intervention components, and feasibility and acceptability of outcome measures). The full “Go, Amend, and Stop” criteria are presented in Table 6.

**Table 6:** Go, Amend, and Stop criteria.

Criterion	Go—proceed with RCT^a^	Amend—proceed with changes	Stop—do not proceed unless changes are possible	Study results	Planned amendments
Usability of digital technology	SUSb score >68 Technology and messages judged highly usable	SUS score ≥63 Technology and messages judged usable	SUS score <52 Technology and messages judged possibly usable	SUS score=72.1 Digital technology judged highly usable	—^c^
Acceptability of intervention components	Intervention components judged highly acceptable	Intervention components judged acceptable	Intervention components judged possibly acceptable	Goal setting and self-monitoring: high acceptability Education: high acceptability Feedback: low acceptability	Feedback: redesign to improve engagement by tailoring message content to actual performance Feedback: enable 2-way communication Feedback: target ≥70% of participants rating feedback as acceptable in the next phase Education: redesign to improve engagement through push notifications and synchronous delivery formats
Feasibility and acceptability of outcome measures	Data completeness ≥95% Outcome measures judged highly acceptable	Data completeness ≥90% Outcome measures judged acceptable	Data completeness <90% Outcome measures judged possibly acceptable	Data completeness: 92.6% at T1d and 90.6% at T2e Outcome measures judged acceptable	Review the Cardiac Self-Efficacy Scale for suitability and consider replacement Enhance questionnaire completion through reminders and brief follow-up support Review all outcome measures with the Public and Patient Involvement panel to identify unclear or burdensome items Target ≥95% data completeness in the next phase

^a^RCT: randomized controlled trial.

^b^SUS: system usability scale.

^c^Not applicable.

^d^T1: baseline.

^e^T2: end-of-study follow-up.

## Discussion

### Overview

This study used a mixed methods approach to assess the feasibility and acceptability of implementing the procedures of a factorial design and delivering multiple intervention components within a digital CR intervention. Importantly, this study was not designed to estimate the main or interaction effects of intervention components but rather to assess the feasibility of implementing these procedures within a factorial design. Findings indicated that the usability of the digital technology met the “Go” progression criteria, while the feasibility and acceptability of the intervention components and outcome measures met the “Amend” threshold. This suggests that modifications to certain components and measures are needed before progressing to an optimization RCT.

Overall, the digital technology used to deliver the intervention was rated as both usable and acceptable. However, feedback on the individual intervention components was more nuanced. The goal setting and self-monitoring component was particularly well received, with participants reporting high usability and acceptability. Engagement data supported these reports, as participants wore the smartwatch for a mean of 41.5 (SD 6.5) days and recorded blood pressure for a mean of 14.3 (SD 13.4) days. Weight was recorded less frequently (mean 3.8, SD 3.1 days), which may be partially due to the absence of a dedicated weighing device. Qualitative findings reinforced the quantitative data, though some participants found the BPM Connect blood pressure monitor uncomfortable and experienced difficulties with wireless data transfer. These challenges are consistent with previous studies identifying wireless blood pressure monitors as prone to technical faults and data synchronization issues [43,44]. Future trials may benefit from routine data checks to identify issues early and ensure accurate and complete data capture.

The high usability and acceptability observed in this study may be understood in the context of existing research on digital health interventions. Usability testing and user-centered design approaches are important for ensuring that digital interventions are effective, efficient, and acceptable to users [45,46]. In addition, technology acceptance models highlight the importance of factors such as perceived usefulness, ease of use, mobile health literacy, and individual characteristics in influencing the acceptability of mobile health apps [47]. In this study, the high usability ratings may reflect the prior development and refinement of the platform, while the generally positive acceptability findings suggest that the intervention was broadly aligned with participants’ needs and capabilities.

The education component was generally regarded as acceptable. Participants described the materials as informative and appreciated the app-based format. However, actual engagement was low, with participants accessing educational materials on a median of only 2.5 (IQR 1.5-3.5) days and viewing a median of 3.5 (IQR 2.5-12) distinct items. Similar patterns of low engagement with digital educational content have been reported in previous studies. For example, Pfaeffli Dale et al [23] found that visits to a CR educational website varied widely, with a median of only 3 visits over 6 months. This highlights the limitations of delivering education solely through a static content library. Options to improve engagement could include providing prompts for participants who are not engaging with materials [48] or adopting alternative delivery methods, such as synchronous group sessions via teleconferencing [43,49]. Previous qualitative studies have identified reduced peer interaction as a drawback of digital CR [14,50], and providing opportunities for group-based learning may help address this. A blended model combining synchronous and asynchronous options could offer patients more flexibility and choice [43], potentially increasing engagement, knowledge acquisition, and behavior change. These findings suggest that the education component should be redesigned to better support these outcomes.

In contrast, the feedback component, delivered via SMS text messages, was the least acceptable to participants of the 3 components. Although none opted out of receiving the messages, many described them as irrelevant, unnecessary, or even irritating. Participants indicated that feedback would have been more useful if personalized and based on their actual behavior rather than generic in tone. One participant suggested that the ability to engage in 2-way communication would have improved the experience, allowing for clarification and support. These preferences align with findings from Gallegos-Rejas et al [51], whose realist synthesis of telehealth-delivered CR identified personalization and shared goal setting as key mechanisms for improving engagement. The absence of personalized feedback in this study was intentional, as the factorial design aimed to isolate the individual effects of each component. However, qualitative findings suggest that feedback without context may have limited value. It may be more beneficial to deliver feedback in combination with goal setting and self-monitoring, allowing intervention providers to tailor feedback to individual behavior and preferences.

The completeness of outcome data was relatively high (>90%); however, some measures had higher rates of missing data. The Cardiac Self-Efficacy Scale had the highest proportion of missing responses (15.4% at baseline and 34.6% at follow-up), suggesting that an alternative measure may be more appropriate for this population. Some participants also encountered difficulties completing the online questionnaires and were offered a paper-based questionnaire instead. This highlights the importance of offering flexible data collection options to suit participant preferences. All other study procedures, including the frequency of contact, the initial face-to-face meeting, and outcome measures, were reported as acceptable by participants.

### Strengths and Limitations

A key strength of this study is its novelty; to our knowledge, this is the first time a factorial trial design has been used to assess a CR intervention. This contributes to the existing literature and demonstrates the potential feasibility of conducting such a study in this context. Another key strength is the use of a mixed methods approach, which provides a comprehensive assessment of feasibility and acceptability. The collection of quantitative and qualitative data offered a richer understanding of participant experiences and highlighted areas for refinement. The use of validated frameworks, including the SUS for usability and the TFA, also strengthened the study’s methodological rigor.

However, several limitations should be noted. First, the small sample size limits the generalizability of the findings. While sufficient to test study procedures, a larger sample with multiple participants per experimental condition will be necessary to accurately estimate engagement, retention, and variability in a full-scale trial. Findings should therefore be interpreted as preliminary and indicative rather than definitive. Second, the use of a convenience sample of also limits generalizability. All participants were White and retired (8/8, 100%), and the majority had some university experience (5/8, 63%), had experienced their cardiac event more than 2 years earlier, and had already completed Phase III rehabilitation (7/8, 87.5%). This profile is likely to be more health literate, experienced with CR, and more motivated than the broader population of patients eligible for CR. There is also potential for self-selection bias, as participants who actively attended Phase IV CR may have had higher motivation than a typical Phase III CR patient. Future work should seek to recruit a more diverse sample to improve generalizability. A further limitation is that the digital intervention was delivered alongside Phase IV CR rather than as a standalone program, making it difficult to determine whether the findings reflect the digital components, the existing rehabilitation program, or a combination of both.

### Implications for Future Research

Trials of digital CR interventions have predominantly used traditional RCT designs, which are well suited to evaluating interventions as a whole but provide limited insight into which components are most effective and how they produce change. To develop more targeted, efficient, and cost-effective interventions, it is essential to understand not only what works but also why and for whom. Isolating and evaluating individual components enables researchers to identify the most impactful components, remove those that are less effective, and streamline intervention delivery while preserving or enhancing patient outcomes.

This approach aligns with the logic of MOST, which advocates for the use of optimization trials to empirically test the performance of individual intervention components. An optimization RCT would be instrumental in identifying which elements of a digital CR intervention meaningfully contribute to behavior change and improved outcomes. Findings from this study suggest that an optimization RCT is potentially feasible and acceptable. However, several uncertainties remain, particularly regarding participant recruitment, retention, and engagement with specific components. Given the low fidelity observed for the education component and the mixed acceptability of the feedback component, targeted refinement of these components is recommended prior to a pilot optimization RCT. Specifically, the education component should be redesigned to improve engagement, for example, through push notifications or synchronous delivery. The feedback component should be evaluated for opportunities for personalization, linking messages to participants’ own behavior data, and for 2-way communication. This phased approach is consistent with the iterative process of the MOST preparation phase. Following this component refinement, a next step could be to conduct a pilot optimization RCT, in which a future trial (or selected parts of it) is delivered on a smaller scale [52]. Although not powered to detect effectiveness, such a pilot would allow researchers to refine the design, study procedures, and intervention components prior to proceeding to a fully powered optimization RCT.

Consideration should also be given to the optimal delivery model for digital CR interventions. While they are often evaluated as standalone alternatives to center-based programs, digital CR can also be implemented in a hybrid format. This was the case in this study, where the digital intervention was delivered alongside Phase IV CR exercise classes. Future optimization research should explore whether digital CR is most effective as a supplement or an alternative model.

### Conclusion

This study demonstrated the feasibility and acceptability of implementing a factorial design and delivering multiple intervention components within a digital CR intervention. The digital technology used to deliver the intervention was rated as highly usable, and the intervention components were broadly acceptable. However, both the education and feedback components require refinement to improve engagement and relevance. These findings highlight the value of the preparation phase of MOST in identifying key areas for improvement before proceeding to full-scale evaluation.

This study also demonstrates the feasibility of implementing the procedures of a factorial design, including randomization across 8 experimental conditions and delivery of the intervention components as intended. These findings suggest that further refinement of intervention components is warranted prior to conducting a pilot optimization RCT to address remaining uncertainties and guide the development of a fully powered optimization trial.

## Appendix

Multimedia Appendix 1 CONSORT 2010 checklist.

Multimedia Appendix 2 TIDieR checklist.

Multimedia Appendix 3 MOST and PREP-REP checklists.

Multimedia Appendix 4 Example SMS text messages.

## Abbreviations

BCT: behavior change technique
COM-B: Capability, Opportunity, Motivation, and Behavior
CONSORT: Consolidated Standards of Reporting Trials
CR: cardiac rehabilitation
CVD: cardiovascular disease
MOST PREP-REP: MOST Preparation Reporting
MOST: Multiphase Optimization Strategy
RCT: randomized controlled trial
SUS: System Usability Scale
TDF: Theoretical Domains Framework
TFA: Theoretical Framework of Acceptability
TIDieR: Template for Intervention Description and Replication
