# Supplementary material for: Optimizing Digital Cardiac Rehabilitation Using the Multiphase Optimization Strategy: Mixed Methods Feasibility Study
Source: JMIR Form Res. 2026 Jun 9;10:e77742. doi: 10.2196/77742 (PMC13291730; doi:10.2196/77742)
Supplement: Multimedia Appendix 2 [file formative_v10i1e77742_app2.docx]

Table S2. Template for Intervention Description and Reporting (TIDieR) Checklist

| Item | Description |
| --- | --- |
| Brief name | The OsCaR (Optimising digital Cardiac Rehabilitation) Study |
| Why | The digital intervention has been developed based on the Capability, Opportunity, Motivation (COM-B) model and the Theoretical Domains Framework.  *Goal setting and self-monitoring component:*  Goal setting and self-monitoring are key strategies used for behaviour modification, as they can empower patients to take responsibility for their health and health-related behaviours. The goal setting and self-monitoring component is designed to assist participants in creating clear mental representations of their desired outcomes while also providing a way of objectively managing their actions toward the attainment of their goals. It is hypothesised that this will increase participants' capability of performing health related behaviours (e.g., physical activity, healthy eating, and medication adherence).  *Education component:*  The education component aims to enhance participants’ knowledge and beliefs about the consequences of their behaviour in the context of cardiovascular disease. It is hypothesised that targeting these theoretical domains will enhance participants’ perceived capability and motivation to improve health-related behaviours (e.g., physical activity, healthy eating, medication adherence, and smoking cessation).  *Feedback component:*  Feedback can play an important role in reinforcing positive behaviours and can provide participants with a valuable means of gauging their progress. The SMS messages aim to enhance participants’ self-efficacy by providing encouragement and guidance. It is expected that the messages will increase participants’ motivation to perform health protective behaviours (e.g., physical activity, healthy eating, and medication adherence). |
| What (materials) | *Goal setting and self-monitoring component:*  Participants will receive the ProACT CareApp, Withings Health Mate app, Withings ScanWatch, and Withings BPM Connect. They will also receive a training manual that describes how to use the ProACT CareApp and digital devices. This component includes the following BCTs: 1.1 goal setting (behaviour), 1.3 goal setting (outcome), 1.5 review behaviour goal(s), 1.7 review outcome goal(s), 2.3 self-monitoring of behaviour, 2.4 self-monitoring of outcome(s) of behaviour, and 2.6 biofeedback.  *Education component:*  Participants will receive the ProACT CareApp and a training manual on how to use it. The education component contains 133 articles or videos. The materials cover the aetiology of cardiovascular conditions (e.g., angina, atherosclerosis, hypertension) and provide tips about cardiovascular lifestyle and medical management. The materials were sourced from the Irish Heart Foundation, the British Heart Foundation, and the Croí Heart and Stroke Charity. This component includes the BCTs: 4.1 instruction on how to perform the behaviour, and 5.1 information about health consequences.  *Feedback component:*  Participants will receive 18 SMS messages over six weeks (3 per week). The messages were designed based on previous studies that aimed to promote behaviour change among patients attending cardiac rehabilitation. Each message targets either physical activity, healthy eating, or medication adherence and contains one of seven BCTs: 1.3 goal setting (outcome), 1.4 action planning, 2.2 feedback on behaviour, 7.1 prompts/cues, 8.3 habit formation, 15.1 verbal persuasion about capability, and 15.3 focus on past success. The messages will be tailored to include the participants' names. |
| What (procedures) | *Goal setting and self-monitoring component:*  An initial face-to-face introductory session will be organised with participants to provide them with the digital technology (e.g., ProACT CareApp, the Withings Health Mate app, the Withings ScanWatch, and the Withings BPM Connect) and training on its usage.  Participants will be instructed to set behavioural goals (e.g., physical activity targets) and outcome goals (e.g., target weight) via the ProACT CareApp. They will use the Withings ScanWatch to track their heart rate and physical activity, and the Withings BPM Connect to monitor their blood pressure. The devices wirelessly transmit readings via Bluetooth to the Withings Health Mate app, which is then synced to the ProACT CareApp. Participants will be able to view and monitor their data on the ProACT CareApp and can review and change their goals at any time.  *Education component:*  An initial face-to-face introductory session will be organised with participants to provide them with the ProACT CareApp. The educational materials will be pre-loaded on the app and will be available to view at participants' discretion.  *Feedback component:*  Participants will receive three SMS messages per week for the duration of the six-week study period. Messages will be tailored to include the participants' names but otherwise will be standardised and sent in a set order. Participants will be able to opt of receiving the messages at any time by replying 'Stop'. |
| Who provided | *Goal setting and self-monitoring component, and education component:*  The introductory session will be delivered by a health psychologist (EK) who is completing this research as part of a PhD. He has experience dealing with patients with cardiovascular disease and will be trained and familiar with the ProACT platform and telemonitoring devices.  *Feedback component:*  The SMS messages will be sent to participants' phones by EK. |
| How (mode of delivery; individual or group) | *Goal setting and self-monitoring component:*  The introductory session will be conducted individually, face-to-face. Participants will use the ProACT CareApp to set and review behavioural and outcome goals; telemonitoring devices (e.g., Withings ScanWatch, Withings BPM Connect) will be used to monitor performance.  *Education component:*  The introductory session will be conducted individually, face-to-face. The educational materials will be delivered to participants via the ProACT CareApp.  *Feedback component:*  The SMS messages will be sent to participants using Textmagic, a text messaging service. |
| Where | *Goal setting and self-monitoring component, and education component:*  The face-to-face introductory session will be held at the Croí Heart and Stroke Charity where participants will be attending exercise classes. Participants will be able to use the ProACT CareApp wherever they choose.  *Feedback component:*  SMS messages will be delivered to participants' smartphones. No specific location is specified. |
| When and how much | *Goal setting and self-monitoring component:*  Participants will attend one face-to-face introductory session. They will be instructed to set activity and weight goals at this session and will be able to review and change their goals as often as they wish. They will be advised to monitor their progress as frequently as they wish.  *Education component:*  Participants will attend one face-to-face introductory session before commencing the intervention. The educational material will be available in a static library on the ProACT CareApp. Participants will be able to view the materials at their discretion.  *Feedback component:*  Participants will receive 18 SMS messages in total, three messages per week. |
| Tailoring | *Goal setting and self-monitoring component:*  Participants will be instructed to set their own personal activity and weight goals and can review and change their goals if they want.  *Education component:*  N/A  *Feedback component:*  SMS messages will be tailored to include the participants' names. The messages will otherwise all be identical and sent in the same order. |
| How well (planned) | *Goal setting and self-monitoring component:*  The number of readings inputted (activity, heart rate, blood pressure) will be recorded by the ProACT platform.  *Education component:*  Participant engagement with the educational materials will be recorded by the ProACT platform. This data will include the number of materials viewed, and time spent viewing the materials.  *Feedback component:*  The successful delivery and receipt of the SMS messages will be recorded, as will the number of participants who choose to opt out of receiving the text messages.  Semi-structured interviews will be conducted to assess the fidelity of receipt and enactment of each component. |
