# Supplementary material for: Optimizing Digital Cardiac Rehabilitation Using the Multiphase Optimization Strategy: Mixed Methods Feasibility Study
Source: JMIR Form Res. 2026 Jun 9;10:e77742. doi: 10.2196/77742 (PMC13291730; doi:10.2196/77742)
Supplement: Multimedia Appendix 3 [file formative_v10i1e77742_app3.docx]

Table S3. The MOST PREP-REP checklist

|  | Item # | Recommendations | Lines |
| --- | --- | --- | --- |
| Introduce the MOST framework | 1 | 1. Identify the use of the multiphase optimization strategy (MOST) with appropriate citation (s) 2. Label aspects of current or prior work that the research team considers preparation for an optimization phase as the preparation phase | 26-28    39-44 |
| Describe the research series | 2 | - 1. Provide context for the current work in the introduction or background and clearly label prior, ongoing, or planned work as preparation where appropriate   2. Describe the trajectory of the research program leading up to the current work.   2.2.a Provide citations for any prior or associated work whether it was completed by the research team or by the others (e.g., literature reviews, formative research, pilot studies, trials)  2.2.b Connect the trajectory of the research program to the MOST phase(s) by labelling prior and current work with the appropriate phase (e.g., preparation phase, optimization phase) including any iterative movement between phases  2.2.c If space is available, provide example(s) of how prior work was translated into elements of the conceptual model and/ or the optimization objective | 39-44  39-44  39-51  39-44 |
| Clearly define the conceptual model | 3 | - 1. Use the term conceptual model to identify the theory or framework guiding the selection of the components, mediators, and outcomes for optimization.   2. Provide a detailed description of the conceptual model including a figure or diagram   3.2.a Identify and describe the intervention components and levels  3.2.b Identify primary (and secondary) outcomes being targeted by the intervention components including description of assessment tools  3.2.c Describe how each intervention component is purported to affect each outcome including descriptions of how relevant mediators will be assessed | 106-107  Figure 1  109-145  162-184  109-145; 162-184 |
| Optimization Objective | 4 | - 1. Use the term optimization objective to identify the factors that will guide the selection of intervention components after the optimization trial   2. Provide an operational definition of the optimization objective   4.2.a Include specific criteria, if known, for determining which components will be retained in optimized intervention such as benchmarks related to effectiveness, scalability, and/or efficiency.   - 1. Describe the rationale for the selection of the optimization objective | N/A |
