# Supplementary material for: Optimizing Digital Cardiac Rehabilitation Using the Multiphase Optimization Strategy: Mixed Methods Feasibility Study
Source: JMIR Form Res. 2026 Jun 9;10:e77742. doi: 10.2196/77742 (PMC13291730; doi:10.2196/77742)
Supplement: Multimedia Appendix 4 [file formative_v10i1e77742_app4.docx]

Table S4. Example SMS Messages

| Behaviour Change Technique | Example message |
| --- | --- |
| 7.1 Prompts/cues | Hi (name), have you gone for a walk today? Lace up and enjoy the fresh air **👟** |
| 15.1 Verbal persuasion about capability | Hi (name), healthy eating gets easier with each decision you make. Start with small changes and build from there. You can do it. |
| 2.2 Feedback on behaviour | Hi (name), you've been consistently active for a week now! That's fantastic progress. Try to keep it up this week. |
| 1.3 Goal setting (outcome) | Balance is everything. Try to fill half your plate with vegetables at every meal. It's an easy guide to eating well. |
| 15.3 Focus on past success | Becoming regularly active is a gradual process. Give yourself credit for what you've done so far! Keep moving and you will achieve all you set out to do! |
| 8.7 Graded tasks | Hi (name), it's week 3! Time to increase the length of your walk. Try adding a few more minutes this week. |
| 5.1 Information about health consequence | Remember, your medication is there to help. Sticking with your medication schedule is key to maintaining a healthy heart ❤️ |
| 1.4 Action planning | Think about how you can fit physical activity into your week. Planning helps make it a reality. You can do it! |
| 8.3 Habit formation | Make sure to take your heart medication. Keeping a consistent routine is important for your health. |
